# The emotional valence of a conflict: implications from synesthesia

**DOI:** 10.3389/fpsyg.2013.00978

**Published:** 2013-12-26

**Authors:** Amit Perry, Avishai Henik

**Affiliations:** ^1^Department of Psychology, Ben-Gurion University of the NegevBeer-Sheva, Israel; ^2^Zlotowski Center for Neuroscience, Ben-Gurion University of the NegevBeer-Sheva, Israel

**Keywords:** synesthesia, conflict, emotional valence, emotional coherency, Stroop-like task

## Abstract

According to some synesthetes’ reports, their experience involves an emotional sensation in which a conflict between the photism and presented color of a stimulus may evoke a feeling of discomfort. In order to investigate the impact of this experience on performance, two experiments were carried out on two synesthetes and their matched control groups. Experiments were tailored for each synesthete according to her unique photism. Participants were presented with stimuli (numerals or words) in colors and were asked to name the color of the stimulus and to ignore its meaning. Incongruent colors were associated with negative or positive emotional words or with non-emotional words. Not surprisingly, an incongruent color (e.g., 5 presented in yellow to a synesthete that sees 5 in red) slowed down color naming. Conflict situations (e.g., a numeral in an incongruent color) created a negative emotional experience. Most importantly, coherence between a conflict or non-conflict emotional experience and the emotion elicited by the color of the stimulus for a given synesthete modulated performance. In particular, synesthetes were faster in coherent than in incoherent situations. This research contributes to the understanding of emotional experience in synesthesia, and also suggests that synesthesia can be used as an instrument to investigate emotional processes in the wider population.

## INTRODUCTION

The word synesthesia means mixing of the senses. Indeed, in synesthesia certain stimuli (e.g., an achromatic written number) may give rise to a perceptual experience in additional modalities (e.g., color or taste) not normally associated with them (Grossenbacher and Lovelace, 2001). Although synesthesia appears to be a perceptual phenomenon, it has been reported that some synesthetes also exhibit strong emotional reactions in response to sensory discord or harmony regarding their synesthetic experiences (Cytowic and Ommaya, 1989; Ramachandran and Hubbard, 2001). Namely, a stimulus colored incongruently with the synesthetic color (i.e., “photism”; e.g., for a synesthete who perceives yellow following a presentation of the digit 5, 5 colored in blue is an incongruent stimulus) would lead to discomfort, while a stimulus colored in a congruent color (e.g., the digit “5” colored in yellow) would lead to a calming mood.

The cognitive conflict between a photism (in the above example yellow) and presented color (in the above example blue) is referred to as a congruity effect. Many use Stroop (Stroop, 1935) and Stroop-like tasks in order to study cognitive conflict. In the commonly used Stroop task, participants are presented with colored color words (e.g., BLUE written in red) and are asked to name the color in which the word is presented (red) while ignoring the word’s meaning (blue). Participants respond slower to incongruent stimuli (BLUE in red) than to congruent stimuli (RED in red; MacLeod, 1991). Similarly, in the synesthetic version of this task (i.e., “synesthetic Stroop task”), synesthetes are presented with colored graphemes and are asked to name the color of the grapheme while ignoring the grapheme’s meaning. Synesthetes are slower to respond in the incongruent condition than in the congruent condition (Mills, 1999; Mattingley et al., 2001; Dixon et al., 2004; Cohen Kadosh and Henik, 2006).

Research using Stroop-like tasks (not including emotional Stroop tasks) referred only to cognitive aspects of the conflict (MacLeod, 1991). However, there may be an emotional aspect to such conflicts; namely, an incongruent condition may produce uneasiness, resembling the synesthetic discomfort described previously. Botvinick (2007) suggested that the behavioral outcomes of a conflict (e.g., a bias toward the selection of tasks and strategies that minimize the risk of conflict) might be due to its registration as an aversive or negatively reinforcing event by the anterior cingulate cortex (ACC) rather than by its triggering, via the ACC, of compensatory shifts in control. Because synesthetes respond emotionally to a conflict, we used a synesthetic Stroop task to detect the emotional aspects of conflict. This is in line with the suggestion of using synesthesia to further our understanding of neurocognitive processes in the non-synesthetic population (Cohen Kadosh and Henik, 2007).

Synesthetic emotional experience was first examined in a study conducted by Callejas et al. (2007). They found that the perception of an incorrectly colored word (colored in the color wheel opposite to the photism) affected the emotional judgment of MA, a grapheme-color synesthete, such that the valence of an emotional or neutral word, presented in an incongruent color, was rated lower on a 7-point Likert scale than the valence of a word presented in the congruent color. In a second experiment, MA and controls were asked to indicate as fast as possible, whether a colored word was positive or negative. Here the congruity effect interacted with the semantic valence of the word, such that correspondence between the emotional valences of the word and the congruity condition led to faster reaction times (RTs). However, a difference between congruent and incongruent conditions appeared only for positive words.

In a later work done by Hochel et al. (2009), an emotional synesthete – R – described that a feeling of discomfort in response to incongruity between a photism and a presented color arose only when the presented color was not emotionally coherent with his photism. For example, the number 5 is red – a positively valenced color. It does not bother R (in fact, 5 in red has a calming effect on R), nor would it bother R if 5 were printed in another positively valenced color such as purple. Hochel et al. (2009) examined R’s performance during an odd/even decision task. Each trial consisted of a number, colored in white, presented in a colored frame that was either congruent or incongruent with the photism associated with the number. The incongruent color was emotionally coherent or incoherent with the photism’s valence.

Hochel et al.’s (2009) work shows that for a specific emotional synesthete, colors possess emotional valence. Taking into account that colors possess an emotional meaning also for the non-synesthete population (Adams and Osgood, 1973; Valdez and Mehrabian, 1994), one can only assume the enhanced emotionality of colors exists for a wider range of synesthetes who experience their world through colors. In this context, we distinguish between three sources of emotionality that arise as a result of the conflict situation described in the previous studies. As mentioned, the first source arises from the congruity between presented and synesthetic color (i.e., the conflict itself). The second arises from the emotional meaning of the colors, while the third arises from the emotional valence of the stimulus. In order to control the emotional valence of colors in our study, each color was selected according to the unique photism of each synesthete and thus had idiosyncratic meaning.

Both Hochel et al.’s (2009) and Callejas et al.’s (2007) studies have shown that the performance of a synesthete is influenced by a different aspect of emotionality that is aroused by the conflict. However, neither study considered all three sources. Specifically, Callejas et al. (2007) related to the emotion aroused by the incoherence between the photism and the printed color as well as to the word’s valence. However, their selection of colors ignored the possibility that colors possess an emotional valence, resulting, in our opinion, in a lack of an effect for negative words. Similarly, Hochel et al. (2009) reported that numbers do not have any emotional valence for R. However, several photisms (of numbers) were emotional and this might have had some impact on number emotionality. Hence, we suggest that it is hard to determine the actual valence of numbers for R. Not assigning a valence might have introduced uncontrolled variance, which might have reduced the effect.

The current study was designed in order to: (a) further the understanding regarding the emotional experience in synesthesia, and (b) examine emotional aspects of cognitive conflict. The study was conducted on two lexical synesthetes who perceive color in response to the meaning of written or spoken words. Also, a control group of non-synesthetes was matched to the synesthetic subjects. Two experiments using a synesthetic Stroop task were conducted, a week apart from each other.

## EXPERIMENT 1

**FIGURE 1:**
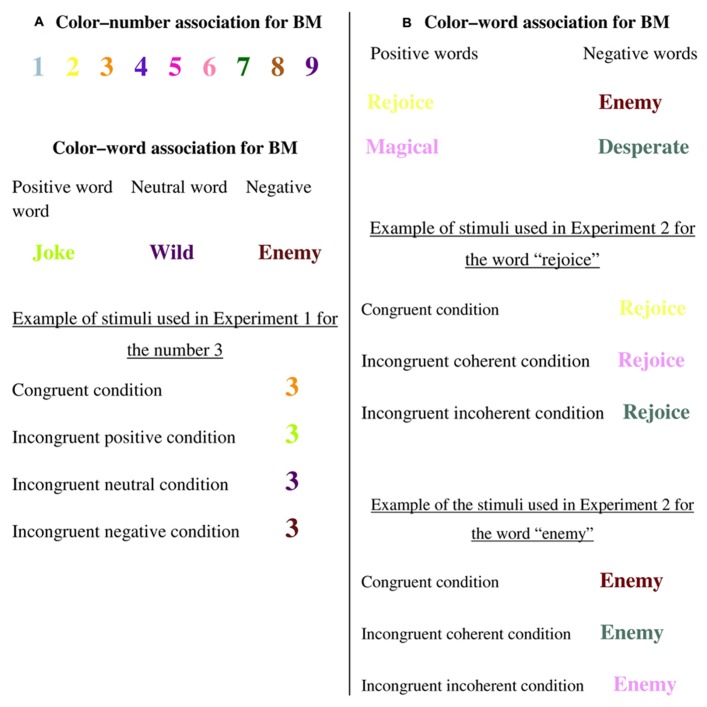
**(A)** Examples of color–number and color–word associations for BM and stimuli in Experiment 1. **(B)** Examples of color–word associations for BM and of stimuli in Experiment 2.

In the first experiment, in order to establish a conflict as being negatively valenced, we aroused only two sources of emotionality by using emotionally neutral stimuli. Because our participants had number–color synesthesia, in addition to their lexical synesthesia, we employed numbers as stimuli since numbers are more neutral than words and this would allow us to use a synesthetic Stroop procedure. The numbers were colored either congruently or incongruently to the synesthetes’ photisms. The incongruent condition (for a given synesthete) was created by using several colors associated with negative or positive emotional words or with non-emotional words (see **Figure 1A**). Namely, we created three incongruent conditions that were similar in conflict between the photism and presented colors but different in their emotional valence (i.e., incongruent positive condition, incongruent negative condition, and incongruent neutral condition).

We expected a two-way interaction between group and congruity. Among synesthetes, we predicted a congruity effect would be found, that is, response to incongruent conditions would be slower than to the congruent condition. More importantly, incongruent conditions would differ from one another – an incongruity effect – reflecting effects of the emotional valence elicited by each of the incongruent conditions. No congruity or incongruity effects were expected for the controls.

## METHODS

### PARTICIPANTS

Participants were selected if their photisms were elicited not just by physical aspects of a word but also by additional conceptual aspects. Two female synesthetes participated in the experiment – AD (23 years old) and BM (20 years old). Both synesthetes had lexical and number–color synesthesia. According to their report, the color of a word was influenced by the word’s meaning and not by the word’s letters. Both synesthetes reported that color could be elicited by different aspects of a word, physical as well as conceptual, but that a word’s emotional valence had a major influence in determining the exact color of the photism. They both said that the appearance of a stimulus in an incompatible color (i.e., a color different from the photism of a given word) elicited a feeling of unease. Also, they indicated that color had some emotional meaning for them. For example, the photism of a name might change due to their familiarization with a person with that name and their feeling toward him/her. In addition, the photism seemed to appear “in their mind’s eye,” floating in a black (BM) or white (AD) background. According to their self-report, the synesthetes had no history of psychiatric or neurological disorders. Both synesthetes were recruited through ads published on the Ben-Gurion University of the Negev web site and on the internet, and both received financial reward for their participation (i.e., 30 NIS – equivalent to ~8.50 USD per hour).

Ten control participants (all females) participated in the experiment, of whom five were matched for each synesthete by age and gender. All of them were students in the Department of Psychology at Ben-Gurion University of the Negev and participated in the experiment as part of a course requirement.

All participants, synesthetes and controls, were Hebrew native speakers, with normal or corrected-to-normal vision and had no diagnosis of attention deficit hyperactivity disorder (ADHD) or dyscalculia.

### STIMULI

**FIGURE 2:**
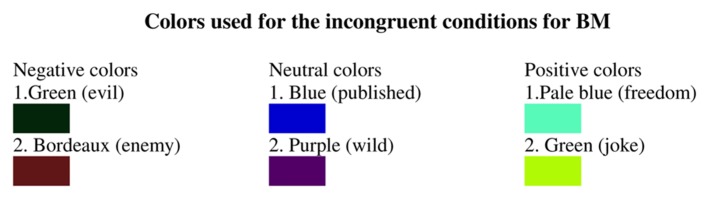
**Colors used for the incongruent conditions (i.e., negative, neutral, and positive) for BM.** The word associated with the color is in parentheses.

Data collection and stimuli presentation were controlled by an IBM Intel Pentium III laptop computer. Stimuli were presented on a DELL E198PF 19-inch LCD monitor. A microphone was placed on a table between the participant and the monitor. Participants were tested individually. They sat approximately 24 inches from the computer screen. The experiment was programmed using E-Prime software. Each trial consisted of presentation of a number from 1 to 9. The number was printed in Times New Roman font, size 144, and presented at the center of a computer screen. For each synesthete and matched control group, each number appeared in a color congruent or incongruent to the synesthete’s unique photism. As mentioned earlier, the incongruent colors were of photisms of emotional and non-emotional words, as reported by the synesthete during the color-matching phase (see Procedure). For each valence (i.e., positive, negative, and neutral) 10 words were selected from the Hebrew lexical norms (Hochman and Henik, 2007). The groups of words were matched in concreteness and familiarity as well as in word-frequency, based on the word-frequency database for printed Hebrew (The Hebrew University of Jerusalem Psychology Department, 2005). Two colors were selected for each incongruent condition. All in all, there were six incongruent colors for each synesthete (see **Figure 2**).

### PROCEDURE

#### Color matching and reliability test

A month before the experiment took place, the synesthetes were asked to report their photisms in response to selected numbers and words. Each color was encoded using red-green-blue (RGB) values in Microsoft Paint. A second color-matching session took place two weeks after the experiment. The settings of the three colors were converted into a single integer value according to the RGB color model by using the formula: RGB value = Red + (Green × 256) + (Blue × 256 × 256). Additionally, the synesthetes were asked to grade the valence of words selected from the Hebrew norms, between 1 (most negative) and 10 (most positive), in order to establish a compatibility between the subjective feeling of the synesthetes and the objective norms.

#### Synesthetic Stroop task

Numbers appeared in color and participants were asked to name the color as quickly and accurately as possible while ignoring the number. There were four conditions – one congruent (e.g., 3 in orange), and three incongruent: incongruent positive (e.g., 3 in the color green associated with the word *joke*), incongruent neutral (e.g., 3 in purple associated with the word *wild*), and incongruent negative (e.g., 3 in bordeaux associated with the word *enemy*; see **Figure 1A**).

Due to the fact that colors used in the experiment uniquely represented each synesthete’s photisms, a color naming block composed of 63 trials was carried out at the beginning of the experiment for fine tuning of color clarification. In this block, in each trial a color appeared on screen and the subjects were asked to name the color as quickly as possible. This enabled them to adjust to color names. Following this, a practice block was carried out, which was made up of 42 trials corresponding to the experimental trials (i.e., colored numbers). Finally, a total of 432 trials were run in six blocks of 72 trials each (18 trials for each condition).

**FIGURE 3:**
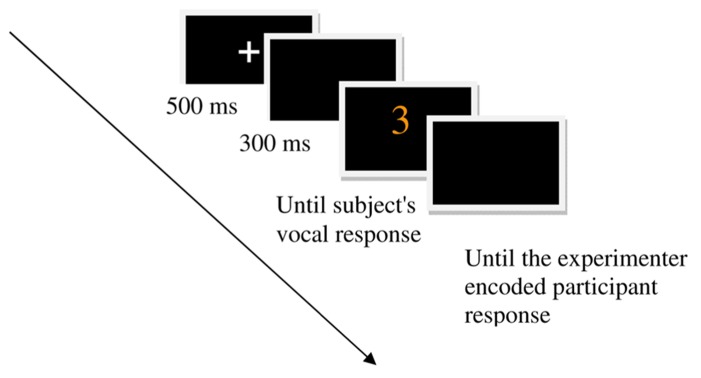
**An example of a congruent experimental trial for BM**. The background color is consistent with BM’s synesthetic experience.

Each trial consisted of a fixation cross (500 ms) followed by a blank screen (300 ms) and then by a colored number display that remained in view until a vocal response was obtained. This was followed by a blank screen until the experimenter encoded the value of the response using a keyboard (**Figure 3**). The background color on the screen was the color in which the photism floated in the mind’s eye of each synesthete – a black background for BM and a white background for AD.

### DESIGN

The variables manipulated were group (synesthetes vs. controls) and congruity (congruent, incongruent negative, incongruent neutral, incongruent positive). Thus, we had a 2 × 4 factorial design with subjects as a random factor. Group was manipulated between subjects, while congruity was manipulated within subjects. The dependent variables were mean RT in milliseconds and accuracy.

## RESULTS AND DISCUSSION

### RELIABILITY TEST

We computed the correlations between the RGB values for the two matching sessions for each synesthete. The colors for numbers had a consistency of 0.99 (*p* < 0.0001) for AD and 0.94 (*p* < 0.0001) for BM. Word colors had a consistency of 0.74 (*p* < 0.001) for AD and 0.78 (*p* < 0.001) for BM. This difference between consistencies may be due to the more frequent introspection (of both synesthetes) of number photisms than word photisms, possibly because of the finite number of single-digit numbers compared to the large number of words. Also, due to their physical and conceptual properties, words are more complicated stimuli than numbers and this may lead to a less stable photism.

### SYNESTHETIC STROOP TASK

#### Accuracy rate

Mean accuracy for both groups was high and ranged between 0.94 and 0.98, implying participants had adjusted to color names during the color naming block. This also indicated that differences between groups could not be explained by speed-accuracy tradeoff. The correlation between mean RTs and accuracy rate was 0.26 for synesthetes (*p* = 0.73) and -0.12 for controls (*p* = 0.88).

#### Reaction time

Incorrect trials, as well as RTs under 150 ms or above 1,500 ms (i.e., 0.7% of correct responses) were excluded from the analysis. Results of the two synesthetes were similar and were grouped.

**FIGURE 4:**
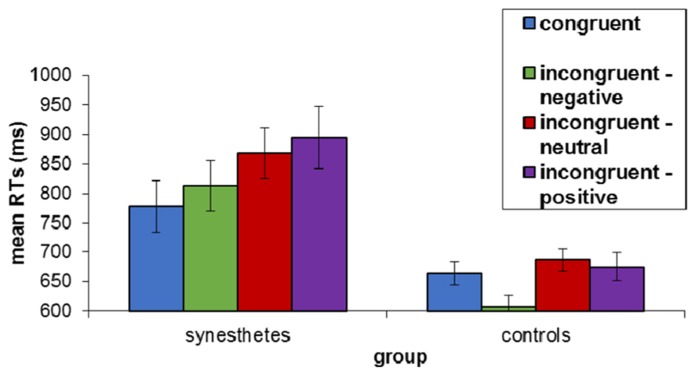
**Mean RT for synesthetes (AD and BM) and control groups in the different congruity conditions (congruent, incongruent negative, incongruent neutral, and incongruent positive) in Experiment 1**. Error bars denote standard error of the mean.

Mean RTs for correct trials of each of the four conditions (one congruent and three incongruent) were subjected to an analysis of variance (ANOVA) with group and congruity as independent variables. We found an interaction between group and congruity, *F*(3,30) = 4.9, *MSE* = 742, *p* < 0.007 (see **Figure 4**). Further analyses showed an effect of congruity for the synesthetes, *F*(3,8) = 7, *MSE* = 742, *p* < 0.01, which was due to faster RTs to the congruent condition than to the incongruent conditions, *F*(1,10) = 7.5, *MSE* = 1,296, *p* < 0.02, similar to what was found in previous studies (Mills, 1999; Mattingley et al., 2001; Dixon et al., 2004). More importantly, there was an incongruity effect, showing gradual changes from the slowest positive incongruent condition, *F*(1,10) = 11, *MSE* = 354, *p* < 0.007, to the fastest negative incongruent condition, *F*(1,10) = 5, *MSE* = 574, *p* < 0.04.

In the introduction we suggested that conflict situations give rise to a negative emotional reaction such as discomfort. Accordingly, the current results show that the incongruent negative condition produced faster RTs than the incongruent neutral or the incongruent positive conditions. Namely, coherence in emotion, elicited by the relation between the conflict and the color emotional association (i.e., negative incongruent condition), led to faster responding. Likewise, incoherence in emotion (i.e., positive incongruent condition) led to slower responding than in the presence of the conflict alone (i.e., incongruent neutral condition). In comparison, Hochel et al. (2009) found no difference between positive and negative incongruent conditions (created by a color frame around the stimulus) for the neutral photism condition (i.e., a digit whose color photism was non-emotional). However, the direction of results was similar to the current results, showing faster RT for the negative incongruent condition.

The controls showed a congruity effect, *F*(3,8) = 18, *MSE* = 742, *p* < 0.001, with the incongruent negative condition being fastest, *F*(1,10) = 4, *MSE* = 805, *p* < 0.001. In addition, the congruent condition did not differ from the neutral and positive incongruent conditions, *F*(1,10) = 1.87, *MSE* = 1,059.34, *ns*, and the neutral incongruent condition and the positive incongruent condition did not differ from each other, *F*(1,10) = 1.78, *MSE* = 361.37, *ns*. Because the incongruent conditions did not produce a conflict in the control group, it is possible that the effect (i.e., incongruent negative condition being fastest) was related to the use of vocal responding rather than motor responding. The first type of responding requires *retrieval* of a color name while the second type only requires recognition of a color name according to the options of response specified. Namely, the particular colors used in the negative incongruent condition were more common colors than other colors in the experiment, so it was easier to recall their names from memory. This suggestion is supported by a similar pattern that was generated in the color naming block.^1^

Finally, a main effect for group was found, *F*(1,10) = 14.5, *MSE* = 14,857, *p* < 0.003, showing that synesthetes responded slower than controls did. This effect can be explained by the synesthetes’ need to ignore not only the stimulus meaning but also its photism (Callejas et al., 2007).

## EXPERIMENT 2

In the second experiment, we employed negatively and positively valenced words as emotional stimuli, adding another source of emotionality (i.e., word meaning) to the color and conflict valences. That way, we could: a) investigate more complex effects of emotional coherence on performance, and b) further investigate a conflict’s negative valence.

As in Experiment 1, the words were colored either congruently or incongruently to their photism. Two incongruent conditions, derived from negative or positive words, served as the color inducers (see **Figure 1B**). For each group of words (i.e., negative or positive), we created an incongruent coherent condition in which valences of the word and incongruent color were emotionally coherent (i.e., a negatively valenced word colored in a negatively valenced word’s photism or a positively valenced word colored in a positively valenced word’s photism), and we also created an incongruent incoherent condition, in which valences of the word and incongruent color were emotionally incoherent (i.e., a negatively valenced word colored in a positively valenced word’s photism or a positively valenced word colored in a negatively valenced word’s photism).

One could hypothesize that among synesthetes, the slowest RT would be found for the incongruent incoherent conditions for positive and negative words. However, coherency between all three sources of emotionality existed only for the negative words in the incongruent coherent condition, while no actual coherent condition was formed for positive words, due to incoherency between word and conflict valence. Thus, we expected an incongruity effect for negative words only.

## METHODS

### PARTICIPANTS

BM, AD, and the control subjects from Experiment 1 participated in Experiment 2.

### STIMULUS

**FIGURE 5:**
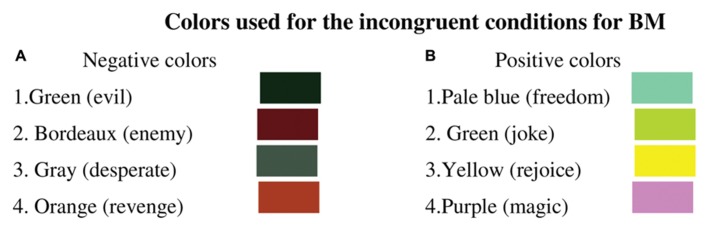
**Colors used for the incongruent conditions for BM**. **(A)** negative colors served as the incongruent colors for the incongruent coherent condition for negative words and the incongruent incoherent condition for positive words. **(B)** Positive colors served as the incongruent colors for the incongruent coherent condition for positive words and the incongruent incoherent condition for negative words. The word associated with the color is in parentheses.

Each trial consisted of a positively or negatively valence word, which constituted an emotional stimulus. The word was printed in Times New Roman font, size 72, and presented at the center of a computer screen. For each synesthete and matched control group, the word appeared in a color congruent or incongruent to the synesthete’s unique photism. The incongruent color represented the photism of a positively or a negatively valenced word, as reported by the synesthete during the color matching phase (see Procedure in Experiment 1). In each of the incongruent conditions, four colors were presented. All in all, there were eight incongruent colors, for each synesthete (**Figure 5**).The specific words that were used in the experiment and from which the incongruent colors were selected, were taken from the same list prepared for Experiment 1. 18 words were selected – nine classified as positive and nine classified as negative.

### PROCEDURE

#### Synesthetic stroop task

Words appeared in color and the subjects were asked to name the color as quickly and accurately as possible while ignoring the meaning. There were three conditions – one congruent (e.g., the word *enemy* in bordeaux), and two incongruent: incongruent coherent (e.g., the word *enemy* in gray associated with the word *desperate*), and incongruent incoherent (e.g., the word *enemy* in purple associated with the word *magical*; see **Figure 1B**).

As in Experiment 1, at the beginning of the experiment participants were presented with a color naming block composed of 78 trials, where they had to name a color that appeared on the screen as quickly as possible, in order to adjust to color names. Afterward, the words were separated into different practice and experimental blocks, according to their valence. For each valence (positive/negative), a practice block of 54 trials was created, corresponding to the experimental trials (i.e., colored words), followed by three experimental blocks, each composed of 108 trials (36 trials for each condition). Valence order was balanced between synesthetes and was identical for each synesthete and her controls. Thus, AD received the positive words blocks first, while BM received the negative words blocks first. All in all, there were 648 experimental trials.

The experiment was held under the same conditions as Experiment 1. Also, the experimental trial was identical to that of Experiment 1 apart from words appearing instead of numbers.

### DESIGN

The variables manipulated were group (synesthetes vs. controls), valence (negative vs. positive), and congruity (congruent, incongruent coherent, incongruent incoherent). Thus, we had a 2 × 2 × 3 factorial design with subjects as the random factor. Group was manipulated between subjects, while valence and congruity were manipulated within subjects. The dependent variables were mean RT and accuracy.

## RESULTS AND DISCUSSION

### ACCURACY RATE

Mean accuracy for both groups was high and ranged between 0.96 and 1.00. As in Experiment 1, this implied that participants had adjusted to color names during the color naming block. The correlation between mean RTs and accuracy rate was 0.0 for synesthetes (*p* = 1.0) and 0.5 for control (*p* = 0.667).

### REACTION TIME

As in Experiment 1, incorrect trials, as well as RTs under 150 ms or above 1,500 ms (0.2% of correct responses), were excluded from the analysis. In addition, results of the two synesthetes were similar and were grouped.

**FIGURE 6:**
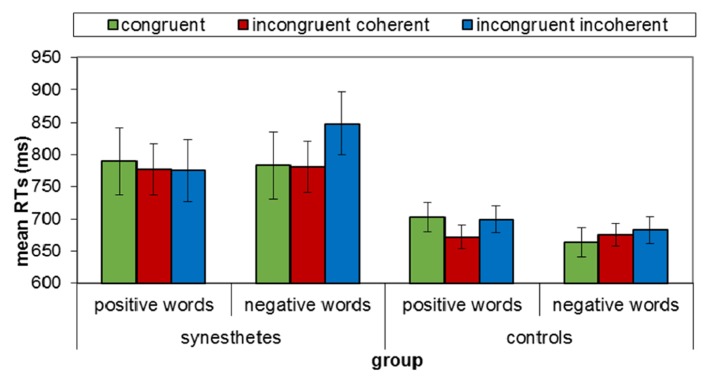
**Mean RT for synesthetes (AD and BM) and control groups for valenced words (positive vs. negative) in the various congruity conditions (congruent, incongruent coherent, incongruent incoherent) in Experiment 2**. Error bars denote standard error of the mean.

Mean RTs for correct trials for each of the six conditions (one congruent and two incongruent for each valence) were subjected to an ANOVA with group, valence, and congruity as independent variables. We found a marginally significant interaction between group, valence, and congruity, *F*(2,40) = 3, *MSE* = 570, *p* < 0.06 (see **Figure 6**).

Further analyses found a significant simple two-way interaction of valence × congruity for synesthetes, *F*(2,4) = 8.8, *MSE* = 213, *p* < 0.0001. This was due to a simple main effect for congruity for negative words, *F*(2,19) = 4, *MSE* = 213, *p* < 0.03, but not for the positive words, *F* < 1. Furthermore, for the negative words, the incongruent incoherent condition was significantly different from the congruent and incongruent coherent conditions, *F*(1,20) = 8, *MSE* = 714, *p* < 0.001, and no difference was found between the congruent condition and the incongruent coherent condition, *F*(1,20) = 0, *MSE* = 425, *ns*. Namely, RT for the incongruent incoherent condition was slower than the other congruity conditions among synesthetes for negative words only.

These results further support the notion that conflict has a negative valence. Thus, coherence in emotion, elicited by conflict, color and valence of a word led to faster responding. Importantly, Hochel et al.’s (2009) report fits in with our results. They found slower RT for positive photisms than for neutral and negative photisms for the negative incongruent condition only.

A significant simple two-way interaction of valence × congruity was also found among controls, *F*(2,36) = 3.6, *MSE* = 610, *p* < 0.03. However, a further analysis showed a simple main effect for positive words, *F*(2,19) = 6.8, *MSE* = 610, *p* < 0.001, but not for the negative words, *F* < 1. The effect for positive words was due to the difference between the incongruent coherent condition and the congruent and incongruent incoherent conditions, *F*(1,20) = 14, *MSE* = 410, *p* < 0.001. No difference was found between the congruent condition and the incongruent incoherent condition, *F*(1,20) = 0.05, *MSE* = 729, *ns*. Namely, RT for the incongruent coherent condition was faster than for the other congruity conditions among controls for positive words only. These results are surprising since for the control group no conflict existed between the photism and the presented color. One could expect that if a difference was found for the control group, it was due to incoherency between word and color emotional valence, resulting from a similar perception of color emotionality for non-synesthetes. However, in this case, the colors used in the incongruent coherent condition were of the same valence as the colors used in the congruent condition, for positive words. As such, no difference had been expected between these conditions.

Finally, a main effect for group was found, *F*(1,20) = 10, *MSE* = 12,061, *p* < 0.001, showing, as in Experiment 1, that synesthetes responded slower than controls did.

## GENERAL DISCUSSION

In summary, both experiments showed an effect of congruity for synesthetes. In Experiment 1, we found a congruity effect showing slower RT for incongruent conditions than for the congruent condition, as well as an incongruity effect showing gradual changes from the slowest positive incongruent condition, to the neutral incongruent condition, until the fastest negative incongruent condition. In Experiment 2, we found the incongruent incoherent condition was the slowest among congruity conditions for negative words only.

Both experiments indicate that emotional coherence influences performance. Namely, faster responses were obtained in the presence of coherence (i.e., incongruent negative condition in Experiment 1; incongruent coherent condition for negative words in Experiment 2) compared to absence of coherence (i.e., incongruent positive condition in Experiment 1; incongruent incoherent condition for positive words in Experiment 2), and to a pure conflict (i.e., incongruent neutral condition in Experiment 1). This pattern is especially interesting in light of the evidence that responding to negative stimuli is usually slower than responding to positive or neutral stimuli (MacLeod and Rutherford, 1992; Fox et al., 2001).

In addition, our results fit in with the assumption that **conflict situations are associated with negative valence**. Major support comes from the finding that RT for the incongruent coherent condition was similar to that of the incongruent incoherent condition for positive words in Experiment 2. The two incongruent conditions differed in their coherency between word and color valence. However, due to the negative valence of conflict, conflict valence and word valence were incoherent in both incongruent conditions, and actually created two incoherent conditions, compared to the congruent condition. These findings also reinforce Botvinick’s (2007) suggestion regarding a conflict’s aversive quality as an explanation for behavioral outcomes in his studies. However, further examination using physiological measures such as galvanic skin response (GSR) is needed in order to establish the emotional differences between experimental conditions.

The ability of an incongruent color to elicit a word whose photism it represents, or at least its emotional valence, supports the assumption regarding the bi-directionality of synesthesia (Cohen Kadosh et al., 2005). Another possible explanation could be that colors possess emotional valence due to fundamental differences in dimensions: hue, saturation, and luminance. Similarly, a connection was found between number quantity size and color saturation and luminance (Cohen Kadosh et al., 2007).

Beyond providing a deeper understanding of the emotional experience involved in synesthesia, our research suggests that similar to exploiting synesthesia to shed further light on our understanding of cognitive processes (Cohen Kadosh and Henik, 2007), it can contribute to our understanding of emotional aspects of human experience. It is important to note that due to difficulty recruiting suitable participants for this research and the fact that only two synesthetes were found to match the study’s criteria, generalization of the current findings to the general population should be done with caution. Importantly, we suggest that in the case of our unique sample of participants, synesthesia enables a glimpse of emotional aspects of cognitive conflict, and how different characteristics of a stimulus, particularly its color, involve an emotional meaning.

## Conflict of Interest Statement

The authors declare that the research was conducted in the absence of any commercial or financial relationships that could be construed as a potential conflict of interest.

## Footnotes

1 This explanation is supported by an ANOVA carried out similarly on the color naming block. It showed that among controls, RT for the colors used in the incongruent negative condition was faster to begin with, *F*(1,9) = 60.94, *MSE* = 621.59, *p* < 0.001. In contrast, among synesthetes, no effect of colors was found in the color naming block, *F*(3,3) = 1.09, *MSE* = 2,264, *ns*.
